# Multimode Switching Broadband Terahertz Metamaterial Absorbing Micro-Devices Based on Graphene and Vanadium Oxide

**DOI:** 10.3390/nano15110867

**Published:** 2025-06-04

**Authors:** Xin Ning, Qianju Song, Zao Yi, Jianguo Zhang, Yougen Yi

**Affiliations:** 1 Joint Laboratory for Extreme Conditions Matter Properties, School of Mathematics and Science, Tianfu Institute of Research and Innovation, Southwest University of Science and Technology, Mianyang 621010, China; nx19130689283@163.com (X.N.); qjsong@swust.edu.cn (Q.S.); 2 School of Chemistry and Chemical Engineering, Jishou University, Jishou 416000, China; 3 Department of Physics and Electronic Engineering, Jinzhong University, Jinzhong 030619, China; 4 College of Physics, Central South University, Changsha 410083, China

**Keywords:** terahertz wideband absorbing micro-devices, metamaterial, multimode switching, graphene, VO_2_

## Abstract

In this paper, we propose a multi-mode switchable ultra-wideband terahertz absorber based on patterned graphene and VO_2_ by designing a graphene pattern composed of a large rectangle rotated 45° in the center and four identical small rectangles in the periphery, as well as a VO_2_ layer pattern composed of four identical rectangular boxes and small rectangles embedded in the dielectric layer. VO_2_ can regulate conductivity via temperature, the Fermi level of graphene depends on the external voltage, and the graphene layer and VO_2_ layer produce resonance responses at different frequencies, resulting in high absorption. The proposed absorption microdevices have three modes: Mode 1 (2.52–4.52 THz), Mode 2 (3.91–9.66 THz), and Mode 3 (2.14–10 THz), which are low-band absorption, high-band absorption, and ultra-wideband absorption. At 2.96 THz in Mode 1, the absorption rate reaches 99.98%; at 8.04 THz in Mode 2, the absorption rate reaches 99.76%; at 5.04 THz in Mode 3, the absorption rate reaches 99.85%; and at 8.4 THz, the absorption rate reaches 99.76%. We explain the absorption mechanism by analyzing the electric field distribution and local plasma resonance, and reveal the high-performance absorption mechanism by using the relative impedance theory. In addition, absorption microdevices have the advantages of polarization insensitivity, incident angle insensitivity, multi-mode switching, ultra-wideband absorption, large manufacturing tolerance, etc., and have potential research and application value in electromagnetic stealth devices, filters and optical switches.

## 1. Introduction

Terahertz technology covers a broad spectrum of application prospects in many fields such as communication, the military, and medicine, and the utilization of metamaterials is important for the future development of terahertz technology [1,2,3].

Metamaterials are a class of artificially designed and manufactured composite materials that do not exist in nature through the design of their internal structures [4,5,6]. These electromagnetic properties make metamaterials potentially useful in radar and sensing technologies, etc. [7,8,9]. Since the end of the 20th century, with progress in nanotechnology and the development of computer simulation tools, the study of metamaterials has gradually emerged and quickly become a hot field of interdisciplinary research. Many achievements have been made so far. For example, the metamaterial structure designed by Rafał Kowerdziej et al. can be effectively tuned at terahertz frequencies, with a transmittance variation of up to 19% [10]. The terahertz metamaterial absorber designed by Yadgar I. Abdulkarim et al. has a strong absorption rate of over 97% at the two absorption peaks of 0.49 THz and 0.69 THz [11]. The use of metamaterials has gradually developed from microwave to infrared and visible light, and high-performance terahertz devices based on metamaterials have also attracted wide attention [12,13]. Many efforts have been made to achieve THz absorber tunability, including the use of dynamically tunable materials such as graphene and the phase-change material VO_2_ [14]. Multispectral terahertz absorbers are crucial for emerging applications such as dynamic electromagnetic stealth, multi-sensing, and optical switching [15]. Among these, the adaptive frequency response is indispensable. Unlike fixed-band devices, multi-mode switchable absorbers can adapt to various terahertz environments in real time, solving the limitations of single-function metamaterials [16,17].

Graphene is a structurally stable material; since it was successfully separated for the first time in 2004, it has quickly become a research hotspot in materials science, physics, chemistry, and other fields, and it has a good response in the terahertz frequency range [18,19,20]. For graphene absorbers, doping or changing the external voltage can regulate the conductivity. These characteristics have led to graphene materials being widely studied for terahertz devices in recent years [21,22,23]. VO_2_ is a transition metal oxide with unique properties, and it is widely applied in the fields of photonics, intelligent regulation and control, and absorbers. The absorber designed by S. Hadi Badri et al., using VO_2_ materials, was able to switch the absorption mechanism from narrowband to wideband; in the narrowband absorption mode, the absorption rate reached 99%, and in the wideband absorption mode, the absorption bandwidth reached 4.88 THz [24]. In addition, other works have revealed the realization of tunable terahertz polarization manipulation devices by combining the phase-change material VO_2_ with a heterogeneous surface design [25]. Phase-change materials have already become a popular research subject [26]. At room temperature, VO_2_ materials have insulating properties, and at a temperature of about 68 °C, they show the properties of a metallic state. Due to this unique property, VO_2_ materials have important research significance in tunable, metamaterial, high-performance devices. In recent years, people have gradually turned their attention to the design of a color absorber that combines two adjustable materials. Many studies have explored the development of an absorber combining graphene and VO_2_ materials that can achieve a variety of different properties. A variety of modes can be achieved during switched absorption, such as wide and narrowband switching absorption, frequency band number conversion, and multiband absorption [27,28,29]. The absorber designed by Zhang et al. can achieve narrowband–wideband mode switching absorption by adjusting the relaxation time of graphene [30]. Gong et al. designed an absorber that can realize tunable switching between two different frequency bands, 0.75–1.15 THz and 2.5–4.5 THz, where regulating the VO_2_ conductivity and the Fermi energy of graphene can both affect its absorption [31]. Tian et al. proposed three-mode switching based on vanadium oxide and a graphene absorber; this enabled the three-mode switching of 2.7–6.2 THz absorption in the low-frequency band, and 5.8–7.6 THz absorption in the high-frequency band and during multi-frequency absorption [32]. Wang et al. designed a switchable absorber, achieving two modes of quadruple narrowband absorption and wideband absorption [33]. However, limitations on the bandwidth of the absorber remain a problem, so it is necessary to propose a terahertz metamaterial absorber with ultra-wideband switching.

In our work, by adjusting the Fermi energy of the graphene material and the conductivity of the VO_2_ material, the absorber is able to switch between three absorption modes; the three modes are: Mode 1 (2.52–4.52 THz), which has an average absorption of 97.67%; Mode 2 (3.91–9.66 THz), which has an average absorption of 95.16%; and Mode 3 (2.14–10 THz), which has an average absorption of 96.07%. All three modes are absorbed over 90% within the absorption range. In addition, we also use impedance matching theory to explain the absorption mechanism, and the absorption of incident light at different incident angles and the absorption of TE wave and TM wave of the absorber are investigated. After calculation and analysis, we found that the structure has little dependence on the low incidence angle, is not affected by the polarization state, and has the features of low angle incidence and being polarization-insensitive. In addition, we also discuss the structural period size and height size, as well as the absorption of the graphene layer pattern size and VO_2_ layer pattern size with different values within a certain range. Finally, we explore the electric field distribution in different modes, and further reveal the absorption mechanism.

## 2. Structural Design and Simulation

**Figure 1:**
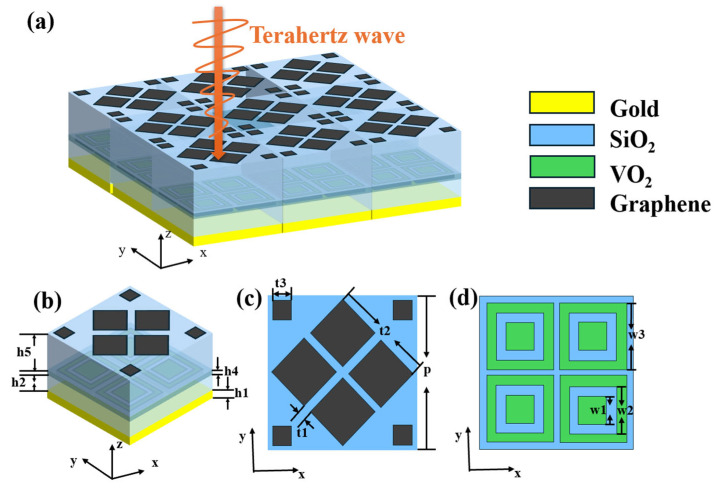
Periodic structure of the proposed MMA (**a**), dimensional parameters of the unit structure (**b**), dimensions of the graphene layer (**c**), dimensions of the VO_2_ layer (**d**).

The periodic structure of the proposed MMA is shown in Figure 1a, and the unit structure is shown in Figure 1b. The structure consists of five layers, namely, the gold substrate, SiO_2_ layer, VO_2_ embedded silica layer, SiO_2_ layer, and graphene layer. After constructing the basic model structure diagram, we used CST simulation software 2020 to simulate the model and adopted the FDID algorithm for simulation and calculations [34,35]. Firstly, periodic boundary conditions are applied on the *X*-axis and *Y*-axis, while the *Z*-axis remains open. Electromagnetic waves propagate along the negative *Z*-axis direction, and the grid size is set as an adaptive grid [36,37]. Based on this design, we can complete the simulation and calculation of the entire periodic array with only one device unit, as shown in Figure 1b. This greatly reduces the calculation time required, speeds up the research process, and maintains the calculation accuracy at the same time.

The thickness of the gold substrate is h1 = 1 μm; this thickness is deep enough to make it fully reflective. The thickness of VO_2_ is h4 = 0.15 μm, the thickness of single-layer graphene is 1 nm, the relative dielectric constant of the dielectric layer material is 2.13, and its thicknesses are h2 = 5.5 μm and h5 = 8.5 μm, respectively. Figure 1c describes the parameters of the graphene layer and Figure 1d describes the parameters of the VO_2_ layer. The middle part of the graphene layer shape is four identical squares rotated 45° in a diamond shape, and the outside is four identical small squares. The embedded VO_2_ layer is a combination of four identical square boxes and a small square placed in the middle. The graphene layer structure model can be fabricated by chemical vapor deposition and graphene transfer techniques, and VO_2_ film can be prepared by magnetron sputtering; by layer-by-layer deposition, the proposed composite structure can be fabricated [38,39]. The parameters were t1 = 1.5 μm, w1 = 3 μm, t2 = 12 μm, w2 = 5 μm, t3 = 4 μm, w3 = 8 μm, and p = 17.5 μm. By adjusting the Fermi energy of the graphene and the conductivity of the VO_2_, the relevant parameters of the three switchable absorption modes of the structure are as shown in Table 1.

The Formula (1) Kubo formula, which can describe the conductivity of graphene [40]; the conductivity can be described by the interband contribution in Formula (2) and the in-band contribution in Formula (3) [41,42]. In the formula, ω, Ef, τ, T, KB, ħ, and e, respectively, represent the frequency of the electromagnetic wave, the Fermi level, carrier relaxation time, temperature, Boltzmann constant, Planck constant, and the basic charge of the electron.

(1) $$\sigma_{g}=\sigma_{intra}+\sigma_{inter}$$

(2) $$\sigma_{intra}=\frac{2k_{B}Te^{2}}{\pi \hbar^{2}}\ln\left(2\cos h\frac{E_{f}}{2K_{B}T}\right)\frac{i}{\omega +i\tau^{-1}}$$

(3) $$\sigma_{inter}=\frac{e^{2}}{4\hbar }\left[H\left(\frac{\omega }{2}\right)+i\frac{4\omega }{\pi }\int_{0}^{\alpha }\frac{H\left(\Omega \right)-H\left(\frac{\omega }{2}\right)}{\omega^{2}-4\Omega^{2}}d\Omega \right]$$

Because we work in the terahertz band, $E_{f}\gg \frac{\hbar \omega }{2}$, and because of the Pauli blocking effect, the band transition’s contribution to graphene’s conductivity is negligible, so its conductivity formula can be simplified to Formula (4) [43]:

(4) $$\sigma_{gra}=\frac{e^{2}E_{f}}{\pi \hbar^{2}}\frac{i}{\left(\omega +\frac{i}{\tau }\right)}$$

It is expressed in Formula (4) that the conductivity of graphene depends on the Fermi energy level, relaxation time, and incident light frequency. In this structure, the electrical conductivity of graphene is mainly regulated by the voltage. Existing experimental studies have shown that in the temperature-change environment required for the switching of graphene between the insulating phase and the metallic phase of VO_2_, the electrical conductivity of graphene does not show significant changes, which is attributed to the counterbalancing effect of phonon scattering and carrier mobility [44,45]. Therefore, within this range, the influence of temperature changes on the electrical conductivity of graphene can be ignored. We can adjust its surface conductivity by doping or changing the external voltage, and we set the relaxation time to 0.1 ps. Electric gate control is a common method for regulating the Fermi level of graphene [46]. Besides this, there are also other alternative methods, such as optical illumination or strong terahertz field excitation, which have been experimentally proven [47,48]. Among these, optical illumination is achieved by irradiating a light source with a photon energy greater than the band gap of the graphene, which can avoid the problem of electrode contamination. As shown in Formula (5), photogenerated carriers are injected into graphene, changing the carrier concentration n, and thereby regulating the Fermi level E_f_. V_F_ is the Fermi velocity and n represents the carrier concentration. The regulation of a strong terahertz field is due to the fact that when a strong terahertz field is incident, the carriers in graphene are driven by the Lorentz force, generating nonlinear drift currents, and thereby achieving dynamic conductivity modulation.

(5) $$E_{f}={\hbar v}_{F}\sqrt{\pi n}$$

The permittivity of VO_2_ can be described by the Drude model and expressed as Formula (6) [31]:

(6) $$\varepsilon \left(\omega \right)=\varepsilon_{\infty }-\frac{{\omega_{p}}^{2}\left(\sigma \right)}{\omega \left(\omega +i\gamma \right)}$$

In the formula, the dielectric constant epsilon $\varepsilon_{\infty }=12$, damped frequency $\gamma =5.75\times 10^{13}rad/s$, and plasma frequency ω_p_(σ) can be represented as ${\omega_{p}}^{2}\left(\sigma \right)=\frac{\sigma }{\sigma_{0}}{\omega_{p}}^{2}\left(\sigma_{0}\right)$, $\omega_{p}\left(\sigma_{0}\right)=1.45\times 10^{15}\;rad/s$, and $\sigma_{0}=3\times 10^{5}S/m$, σ = 3 × 10^5^ S/m. When σ = 2 × 10^2^ S/m, VO_2_ is considered an insulating state with a corresponding temperature of 300 K, and when σ = 2 × 10^5^ S/m, VO_2_ is considered a metallic state with a corresponding temperature of 340 K.

## 3. Results and Discussion

**Figure 2:**
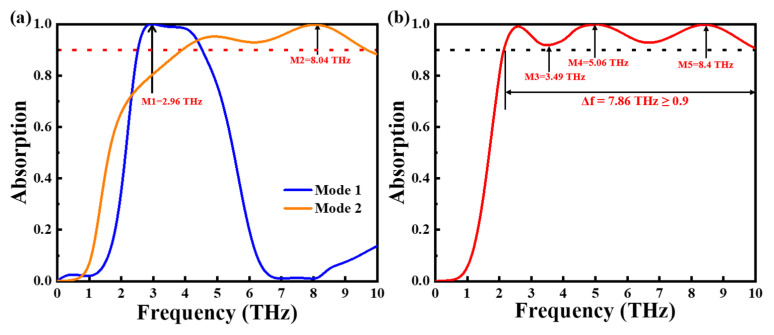
Absorption of Modes 1 and 2 (**a**), absorption of Mode 3 (**b**).

As shown in Figure 2a, in Mode 1, we found that in the bandwidth of 2.52–4.52 THz, the absorption of the absorber could reach 90%, with an average absorption rate of 97.67%, and that the perfect absorption was achieved at the frequency point of 2.96 THz, with an absorption efficiency of 99.98%. For the convenience of subsequent discussion, we defined this frequency point as M1. In Mode 2, where the absorber graphene has a Fermi level of 0.2 eV, a conductivity of 200,000 S/m for VO_2_, and absorbs more than 90% within the frequency band of 3.91–9.66 THz, the average absorption is 95.16%. At the frequency point 8.04 THz, the absorption reaches 99.76%, which we define as M2. At a Fermi energy of 0.7 eV for graphene and a conductivity of 200,000 S/m for VO_2_, the absorber is in absorption Mode 3. Figure 2b describes the absorption rate in this mode. Through the coupling effect, the absorber can realize ultra-wideband absorption of more than 90% in 2.14–10.00 THz; the general absorption efficiency is 96.07% and the general absorption efficiency is 99.85% at the frequency of 5.06 THz, achieving almost perfect absorption. The absorption rate is 99.76% at the frequency of 8.4 THz and 91.83% at the frequency of 3.49 THz. We define 3.49 THz, 5.06 THz, and 8.4 THz as M3, M4, and M5, respectively. The absorption bandwidth in Mode 3 is already higher than that of most broadband absorbers. We have made a detailed comparison and analysis between the absorber proposed and other similar structural models, as shown in Table 2 [49,50,51,52,53,54]. It can be observed that the advantages of the structure designed by us are that it can achieve a higher absorption bandwidth with fewer structural layers and can switch between multiple absorption modes.

For the purpose of examining the absorption features of the structure in greater detail, we introduce the impedance matching principle. The relative impedance is expressed by Formula (7) [55]:

(7) $$Z=\pm \sqrt{\frac{{\left(1+S_{11}\right)}^{2}-{S_{21}}^{2}}{{\left(1-S_{11}\right)}^{2}-{S_{21}}^{2}}}$$

**Figure 3:**
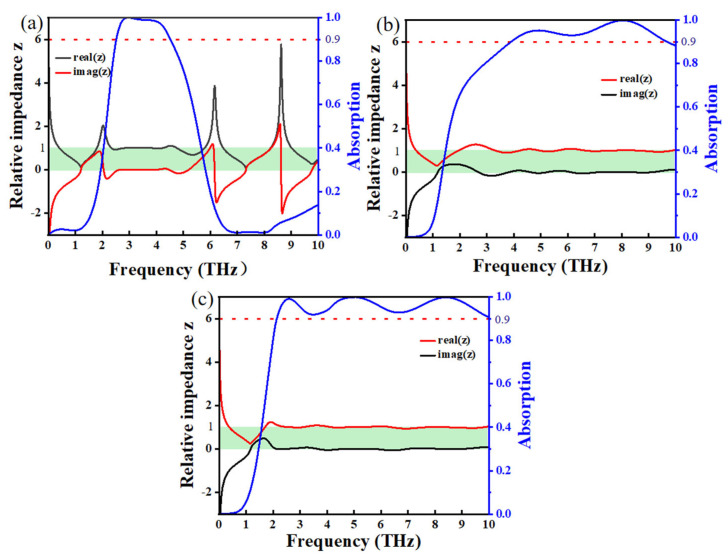
Relative impedance and absorption of Modes 1 (**a**), 2 (**b**), and 3 (**c**).

S_11_ is the reflection coefficient and S_21_ is the scattering coefficient; the reflectivity can be expressed as $R(\omega )={\left|S_{11}\right|}^{2}$ and the transmittance can be expressed as $T(\omega )={\left|S_{21}\right|}^{2}$. We use the formula $A(\omega )=1-R(\omega )-T\left(\omega \right)$ to express the absorption rate, according to the impedance matching theory. When the impedance of the incident electromagnetic wave matches the characteristic impedance of the material, the energy of the electromagnetic wave will enter the material, reducing the reflection and increasing the absorption; at this time, the real part of the complex impedance is almost equal to 1, while its imaginary part is roughly maintained at 0, and electromagnetic waves can be maximized into the material and absorbed [56,57,58,59]. Figure 3a–c shows the impedance in three modes; we found that in the range of 2.52–4.52 THz under Mode 1, 3.91–9.66 under Mode 2, and 2.14–10.00 THz under Mode 3, the real part of the relative impedance approaches 1, while the imaginary part approaches 0. The complex impedance satisfies the impedance matching theory, resulting in high absorption; this theoretically reveals the absorption mechanism of our absorber. In addition, at the frequency points M1 of Mode 1, M2 of Mode 2, and M4 and M5 of Mode 3, the impedance is 1.0000829–0.000118267i, 0.99764128–0.000525042i, 1.0013537–0.000541371i, and 0.99863559–000197135i, which is consistent with the impedance matching theory. This also reveals an absorption rate of more than 99.7% for these points.

Then, we explored the absorption of the absorber Mode 1 and Mode 3 at different Fermi levels, respectively. The Fermi energy of graphene is expressed by Formula (8), where V_g_, ε_0_, ε_r_, and d represent the bias voltage, dielectric constant in a vacuum, relative dielectric constant of the medium, and dielectric layer thickness [60]:

(8) $$E_{f}\approx h\nu_{f}\sqrt{\frac{\pi \varepsilon_{r}\varepsilon_{0}V_{g}}{ed}}$$

**Figure 4:**
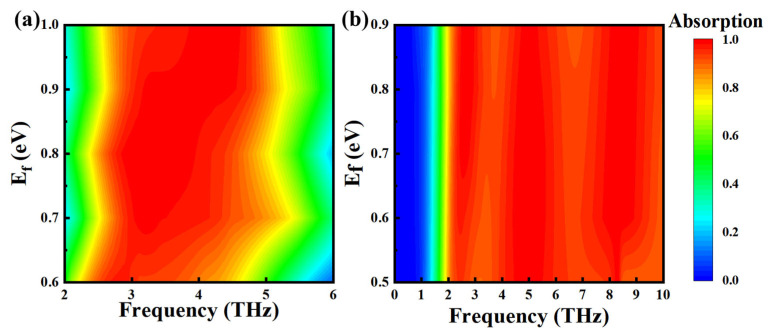
Absorption rate of Mode 1 at different Fermi energies (**a**), Mode 3 at different Fermi energies (**b**).

Figure 4a describes the absorption at Mode 1; in this mode, VO_2_ is in an insulated state, where the Fermi level changes within the range of 0.6 eV to 1.0 eV. It can be found that at 0.6 eV, the absorption bandwidth is 2.47–3.79 THz (1.32 THz), and at 0.8 eV, it is 2.52–4.52 THz (2 THz), and that the absorption bandwidth gradually increases. When the Fermi level continues to increase to 1.0 eV, the absorption bandwidth shows little variation, but the average absorption rate decreases. The average absorption rate was 97.67% at 0.8 eV and 96.67% at 1.0 eV. Figure 4b describes the absorption of the Fermi level of the absorber over the frequency range of 0.5–0.9 eV in Mode 3. The average absorption rate rises initially and then falls; the average absorption rate of 0.5 eV was 93.68%, and that of 0.7 eV and 0.9 eV was 96.07% and 95.33%. When the absorption is greater than or less than 0.7 eV, the absorption shows a small oscillation. At 0.7 eV, absorption is relatively flat; therefore, it can be seen as the effect of the two-absorption coupling. In summary, regardless of whether VO_2_ is in a metallic state or an insulated state, the regulation of the Fermi level of the graphene layer can be used to regulate the absorption.

**Figure 5:**
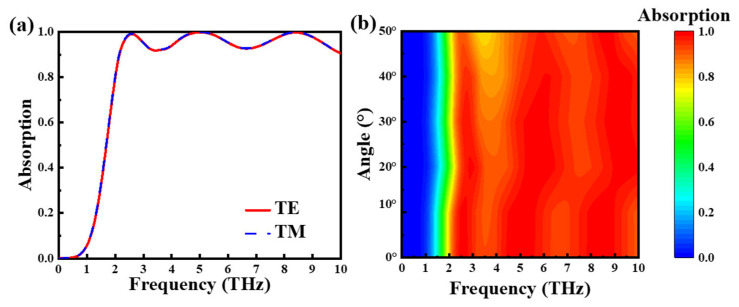
Absorption curves of Mode 3 at TE and TM modes (**a**), and Mode 3 at 0–50° incidence angles (**b**).

We explored the absorption for various polarization states and different incidence angles in the ultra-wideband absorption Mode 3. Figure 5a describes the absorption under a TE wave and TM wave, and it is apparent that the absorption rate is not affected by the polarization state of the incident wave. This is because the graphene layer in our absorber model, as well as the VO_2_ layer pattern, are centrosymmetric. This property enables the absrober to provide consistent performance in different polarization states, which also allows the model to be applied to a wider range of practices [61,62,63]. Similarly, we can see that Figure 5b describes that the absorption rate of incident waves varies between 0 and 50°. The average absorption rate at 0°, 10°, 20°, 30°, and 40° is 96.07%, 95.96%, 95.65%, 94.96%, and 93.38%. Furthermore, the average absorption rate can reach over 90%, which also shows that the structure displays the features of low angle incidence and insensitivity, further expanding the use of the structure in practical applications.

**Figure 6:**
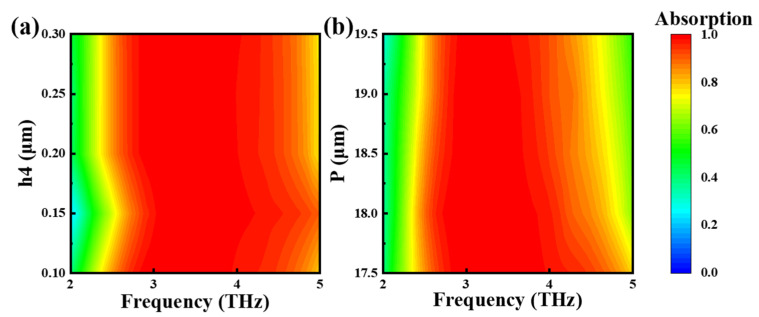
Absorption of Mode 1 at different thicknesses of the VO_2_ layer (**a**), absorption of Mode 1 at different sizes of period p (**b**).

Next, we discuss the absorption of Mode 1 in various sizes of period p and embedded VO_2_ layer thicknesses h4, as well as that of Mode 3 in various sizes period p, dielectric layer thicknesses h2, h4 and embedded VO_2_ layer thicknesses. As shown in Figure 6a, absorption Mode 1 is affected by the thickness of the embedded VO_2_ layer, and the average absorption rate is 96.07% when h4 = 0.15 μm. With increases or decreases in h4, the average absorption rate will decrease, but all rates are above 90%, When h4 was 0.1 μm, 0.2 μm, and 0.3 μm, the average absorption rates were 95.9%, 94.96%, and 92.15%. As shown by Figure 6b, as period p increased, there was a slight change in the absorption bandwidth. When p = 17.5 μm, the absorption range was 7.86 THz, while when p = 19.5 μm, the absorption range was 7.63 THz. Through the analysis of the above parameter period p and the absorption of vanadium oxide layer thickness variation, we can conclude that the structure allows a large manufacturing tolerance, which makes it more feasible for application.

**Figure 7:**
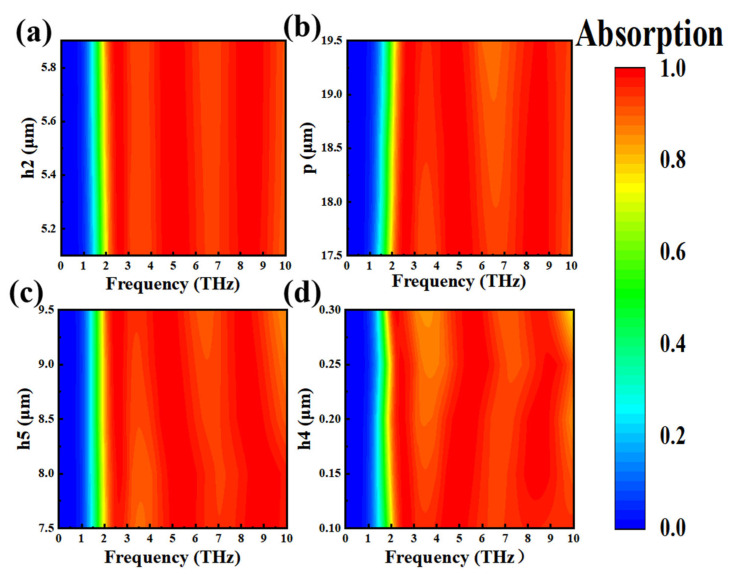
Absorption rate of Mode 3 at different dielectric layer thicknesses h2 (**a**), Periodic p (**b**), h5 (**c**), and VO_2_ layer thicknesses h4 (**d**).

We continue to look at Figure 7a,b, and it can be seen that the thickness of the VO_2_ layer has a very small influence on the absorption of Mode 3, while the influence of period p is large. Along with the growth of period p, the average absorption rate gradually decreases. From Figure 7c, we can find that the average absorption rate of h5 is the highest when the dielectric layer thickness is h5, and that the absorption bandwidth narrows when h5 becomes larger or smaller. In addition, when h5 = 7.5 μm, the absorption rate is less than 90% at 3.28–3.89 THz; the absorption rate of this section clearly decreased. Furthermore, when h5 = 9.5 μm, the absorption bandwidth was 2.1–9.3 THz (7.2 THz); the decrease in the absorption bandwidth was obvious. Therefore, it is concluded that the structure is insensitive to the thickness accuracy of the dielectric layer embedded in the VO_2_ layer, and the optimal values of the parameters p, h4, and h5 are 18.5 μm, 0.15 μm, and 8.5 μm, respectively.

**Figure 8:**
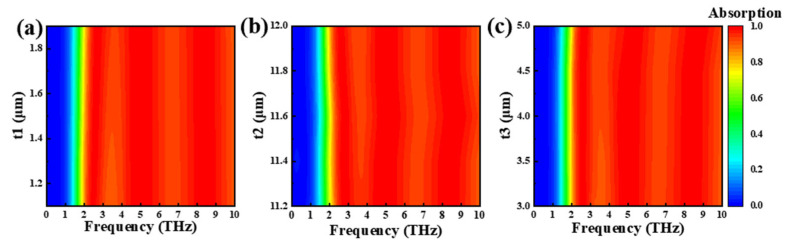
Mode 3 absorption rate at different sizes of graphene layer parameters t1 (**a**), t2 (**b**), t3 (**c**).

Figure 8a–c describes the absorption of graphene layer parameters t1, t2, and t3 at different sizes in Mode 3. We found that the absorption effect of each parameter changed very little within the explored variation range, and that the average absorption rate could always remain above 90%. When the distance t1 between the four squares in the middle of the graphene layer increases, the average absorption of t1 at 1.0 μm and 2 μm is 95.74% and 96.35%, respectively. When the graphene layer parameter t2 is 11.2 μm and 12 μm, the absorption is 96.5% and 96.07%. Similarly, the small square size of the four corners of the graphene layer has little effect on absorption; the average absorption of t3 in 3.0 μm, 4.0 μm, and 5.0 μm was 95.82%, 96.07%, and 96%, respectively. The above parameters were in different sizes, and the absorption rate showed almost no change. This is because graphene-supported plasmon patterns typically concentrate at the edges or defects of a pattern, creating a strong local field enhancement effect. Even if the pattern size changes, the plasmon resonance effect remains significant as long as key areas remain similar. This further shows that the structure allows a large manufacturing tolerance, which is conducive to practical applications [64,65,66].

**Figure 9:**
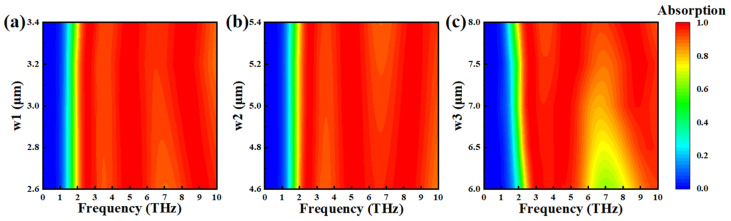
The absorptivity of Mode 3 under VO_2_ layer parameters w1 (**a**), w2 (**b**), w3 (**c**) at different sizes.

Figure 9a describes the impact of changes in the VO_2_ layer parameter w1 on the absorption performance within a certain range. It is found that when w1 increases from 2.6 μm to 3.0 μm, the average absorption rate exhibits a downward trend: at 2.6 μm and 3.0 μm, the average absorption rate is 95.36% and 96.07%, respectively; at 2.6 μm, the absorption is less than 90% and is about 89% at 6.71–7.41 THz. Increases in w1 result in a reduction in the absorption bandwidth, and the absorption bandwidth reaches 7.19 THz when the VO_2_ layer width (w1) is 3.4 μm, but overall, the absorbers show good absorption. Similarly, Figure 9b depicts the absorption of w2 at different values. Our analysis calculated that the average absorption rate was highest when w2 = 5 μm. Figure 9c shows that the parameter w3 exerts a strong effect on the absorber. When the size of w3 increases, the average absorption rate increases significantly and the absorption bandwidth decreases; therefore, the structure is insensitive to the parameters w1 and w2 of the VO_2_ layer, while the optimal size of w3 is 8.0 μm.

**Figure 10:**
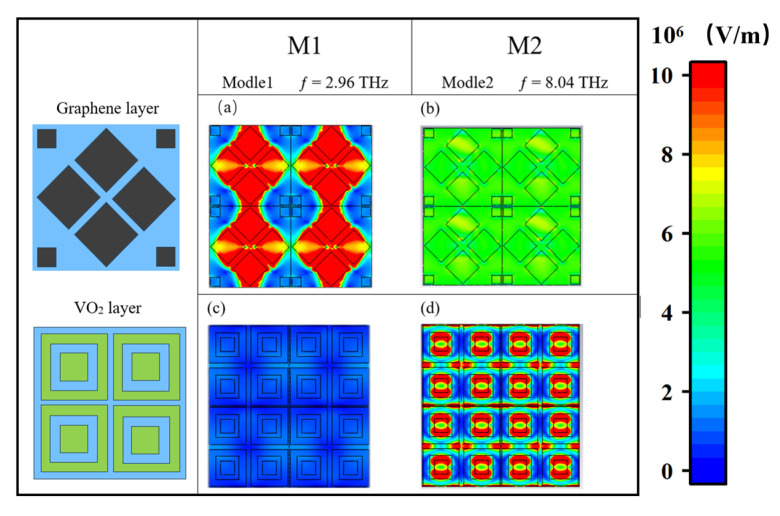
Electric field distribution at Mode 1 point M1 (**a**,**c**), M2 (**b**,**d**).

Then, to further elaborate on the absorption properties, we investigate the distribution of the electric field at the special frequency points M1–M4, as shown in Figure 10a,c. At the M1 frequency point of Mode 1, it is obvious that the electric field is mainly distributed in the middle four squares of the graphene layer, because the surface plasmon resonance [67,68,69] shows a strong intensity in this region. The electric field distribution in this region is highly concentrated, resulting in the absorption efficiency of the electromagnetic wave energy reaching a peak, while the VO_2_ layer has almost no electric field, which is because the VO_2_ in this mode is in an insulating state, and VO_2_ is a thin insulating material sandwiched between two layers of silicon oxide [70]. It forms a relatively uniform dielectric environment with the surrounding silicon oxide layer, which means that the electric field does not change or concentrate significantly in this region. In contrast, the top layer of graphene has extremely high electron mobility and excellent electrical conductivity, which can effectively constrain the electric field near its surface, thus enhancing the concentration of the electric field in this region [71,72,73]. This indicates that Mode 1 is mainly derived from the graphene layer. However, in Mode 2, as demonstrated by Figure 10b,d, the electric field primarily resides on the square frame of the VO_2_ layer, as opposed to the graphene layer where the electric field is relatively reduced, which indicates that Mode 2 is mainly from the VO_2_ layer, because the conductivity increases significantly when VO_2_ becomes a metallic state, at which point it can strongly reflect and absorb incoming electromagnetic waves. When the Fermi level of graphene is low, its internal free carrier concentration is reduced, thereby reducing its response to electromagnetic waves. The structure supports a specific resonance mode, where the VO_2_ layer plays a key role in the electromagnetic coupling effect, forming a strong local field enhancement effect [74,75,76].

**Figure 11:**
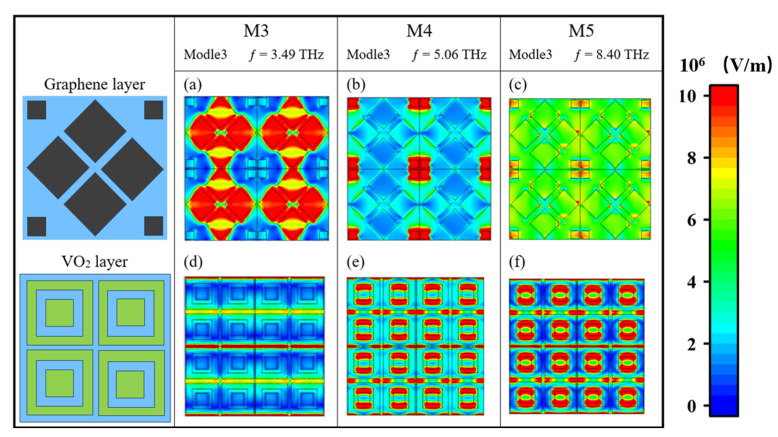
Electric field distribution at Mode 3 point M3 (**a**,**d**), M4 (**b**,**e**), M5 (**c**,**f**).

Finally, we also discussed the arrangement of the electric field of the three special resonance points in Mode 3, as shown in Figure 11a,d. The intense electric field at M3 is mainly distributed in the four square blocks in the middle of the graphene layer, but the strength is not as strong as that at M2, and the low-intensity electric field is dispersed at the boundary of the adjacent unit structure of the VO_2_ layer. The graphene layer contributed more to the absorption at this point. As can be seen from Figure 11b,e, the electric field distribution at point M4 shows that the strong electric field is distributed in both the graphene layer and the VO_2_ layer. In the graphene layer, it is distributed at the four small squares, and in the VO_2_ layer, it is distributed at the junction of the box and the adjacent unit structure, indicating that both the graphene and the VO_2_ contribute greatly to the high absorption there. Looking at the electric field distribution at point M5 in Figure 11c,f, we can clearly see that the strong electric field there is distributed at the junction between the box of the VO_2_ layer and the adjacent unit structure, which is almost the same as the distribution of the VO_2_ at the M4 point; while the electric field distribution of the graphene layer is weak, the VO_2_ layer makes a greater contribution to the absorption at this point. In summary, the whole multi-layer structure acts as a resonator system, and the coupling effect between the layers of materials is generated between them; then, broadband absorption is formed.

## 4. Conclusions

To sum up, we propose an absorber with three tunable modes: Mode 1 (low band absorption mode), Mode 2 (high band absorption mode), and Mode 3 (broadband absorption mode). When the absorber is in Mode 1, the absorber has greater than 90% absorption across the range of 2.52–4.52 THz. When the absorber is in Mode 2, the absorber has greater than 90% absorption across the range of 3.91–9.66 THz. When the absorber is in Mode 3, the absorption at 2.14–10 THz reaches more than 90%. The absorption mechanism was revealed by using the relative impedance theory, and the absorption of incident waves under various polarization states and different incident angles was investigated, which showed that the structure was a polarization-insensitive and low-angle-insensitive structure. The absorption of each parameter in different sizes was also investigated, and the optimal size was obtained after calculation and analysis. Finally, the electric field distribution of special frequency points in different modes was calculated and drawn. The absorption mechanism of the structure was further revealed by the theory of the electromagnetic coupling effect. Overall, this type of metamaterial device has significant application significance in fields such as terahertz communication filtering, electromagnetic stealth, and radar detection. However, it may also encounter some practical issues, such as the need to break through bottlenecks in material regulation compatibility, manufacturing processes, and dynamic response speed. Future research should focus on new regulation mechanisms, process innovation, and intelligent design to promote the practical and integrated development of terahertz metamaterial devices.

## Figures and Tables

**Table 1:** Parameters related to different modes of proposed MMA.

Mode	E_f_ (eV)	σ_VO_2__ (S/m)	f_l_–f_h_ (THz)	Bandwidth (THz)	A_ave_
I	0.8	475	2.52–4.52	2.00 THz	97.67%
II	0.2	200,000	3.91–9.66	5.75 THz	95.16%
III	0.7	200,000	2.14–10.00	7.86 THz	96.07%

**Table 2:** The model is compared with other similar structural models.

References	Absorption Bandwidth (THz) (≥0.9)	Ultra-Wideband (Bandwidth > 7 THz)	Multimodal Tunability
[49]	Multiband (onepeak): 0.745–0.775 broadband: 2.3–5.63	No	Yes
[50]	Multiband (one peak): 2.3 ultra-wideband: 2.85–10	Yes	Yes
[51]	Dual-band absorption: 2.55–4.35 broadband absorption: 7.6–10.85	No	Yes
[52]	Quadruple broadband: 3.58–10 1.90–5.29 5.24–6.19 5.72–8.32 7.33–10.00	Yes	Yes
[53]	2.99–6.16	No	No
[54]	3.7–8.0	No	No
Proposed	triple-band: 2.52–4.52 3.91–9.66 2.14–10.00	Yes	Yes

## Data Availability

Publicly available datasets were analyzed in this study. This data can be found here: [https://www.lumerical.com/] (accessed on 1 January 2020).
